# Short Supply of High Levels of Guanidine Acetic Acid, Alters Ovarian Artery Flow and Improves Intraovarian Blood Perfusion Area Associated with Follicular Growth in Sheep

**DOI:** 10.3390/ani15020143

**Published:** 2025-01-09

**Authors:** Marta da Costa Sousa, Camila Muniz Cavalcanti, Alfredo José Herrera Conde, Bruna Vitória de Freitas Alves, Larissa Fernandes Baia Cesar, Jhennyfe Nobre de Sena, Yohana Huicho Miguel, César Carneiro Linhares Fernandes, Juliana Paula Martins Alves, Dárcio Ítalo Alves Teixeira, Davide Rondina

**Affiliations:** 1 School of Veterinary Medicine, Ceará State University (UECE), Fortaleza 60714-903, Ceará, Brazil; marta.costa@aluno.uece.br (M.d.C.S.); camila.muniz@uece.br (C.M.C.); alfredo.herrera@aluno.uece.br (A.J.H.C.); bru.freitas@aluno.uece.br (B.V.d.F.A.); larissa.baia@aluno.uece.br (L.F.B.C.); jhennyfe.sena@aluno.uece.br (J.N.d.S.); yohana.huicho@aluno.uece.br (Y.H.M.); juli.alves@uece.br (J.P.M.A.); darcio.teixeira@uece.br (D.Í.A.T.); 2 School of Health Sciences, University of Fortaleza, Fortaleza 60811-905, Ceará, Brazil; caancesar@gmail.com

**Keywords:** blood perfusion, creatine, follicle, guanidine acetic acid, ovary, sheep

## Abstract

Guanidinoacetic acid (GAA), a precursor of creatine, has a recognized effect on ruminant performance when used as a dietary supplement. However, its impact on reproductive response remains to be elucidated. Therefore, this study aimed to contribute initially to this area by supplementing the diets of ewes with a high dose of GAA, evaluating its effects on reproductive response. Twenty adult sheep had their estrus synchronized using an MPA sponge, eCG, and PGF2α. After estrus detection ewes were mated. For 10 days until mating, ewes were grouped in groups of baseline diet (BSD; n = 10) and GAA diet (GAAD; n = 10), which was the BSD with daily 0.9 g/kg DM of GAA. After the eCG + PGF2α dose, the GAAD group exhibited an increase in the peak diastolic and pulsatility of the ovarian artery, a reduction in the systolic/diastolic peaks ratio, and a larger intraovarian blood perfusion area. A greater depletion of follicles with <3 mm was observed in the GAAD group and a higher number of follicles ≥3 mm. No differences were observed between the diets respect to pregnancy, and twin rates. Thus, a high GAA supply before mating significantly alters ovarian vasculature and improves follicular growth in ewes but does not affect the pregnancy rate.

## Simple Summary

AAs are crucial for various reproductive functions, including hormone synthesis and follicular development. However, their use in ruminants is limited by ruminal degradation. GAA, a precursor to creatine, offers a promising alternative as it bypasses ruminal breakdown to a significant extent. GAA has shown positive effects on growth performance and feed efficiency in cattle and sheep. While research in this area is still limited, preliminary findings suggest that GAA may improve placental vascularization in cows. Further research is needed to fully understand the effects of GAA on reproductive processes in ruminants. In this study, GAA supplementation was shown to influence ovarian function and follicular development but did not improve overall pregnancy outcomes in ewes.

## 1. Introduction

In ruminants, precise estimates of amino acid (AA) requirements for supporting ovarian response or mating success remain unavailable. This uncertainty stems from the management of dietary protein degradation in the ruminal environment, where the amino acid chains are reorganized to synthesize the walls of ruminal microorganisms, and a portion of the dietary AAs that flows to the small intestine is metabolized and utilized by the individual [1]. Therefore, adopting strategies to increase the flow of AAs to the duodenum is essential. Recent studies have unveiled promising findings, showing that some dietary AAs exhibit relatively lower ruminal degradation compared to others and directly influence ovarian functionality. For example, glutamate, the primary excitatory neurotransmitter of the central nervous system [2], acts through Kiss1 neurons to stimulate the production of GnRH [3]. Intravenous administration of glutamate in goats has been shown to enhance ovarian vascular activity, follicular growth, and ovulation rate [4]. Similarly, dietary supplementation with glutamate in the same species has stimulated feed intake, increased follicular growth, and improved intraovarian blood flow during ovulation [5]. Part of the dietary glutamate that reaches the enterocytes is degraded to produce ATP and synthesize glutathione, an important antioxidant molecule [2], which positively influences GnRH and, consequently, FSH and LH levels [6,7]. Moreover, glutamine, the primary precursor of glutamate, also plays a role in follicular activation through the positive regulation of phosphoinositide 3-kinase, mTOR, and IGF-1 [8,9].

In recent years, significant attention has been focused on the use of AAs in nutritional intervention strategies specifically aimed at optimizing reproductive processes. In this context, recent studies have explored the actions of AAs in humans [10] and animals [11]. These studies highlight the roles of AAs as neurotransmitters in the neuroendocrine system and as precursors to several hormones and neuropeptides, such as the protein kinase mammalian target of rapamycin (mTOR) [12] and estrogen [13]. These compounds are integral to various bodily functions, including energy homeostasis and reproductive activity [1]. Among these roles, dietary AAs regulate the synthesis of insulin-like growth factor 1 (IGF-1), which is involved in the transcriptional activity of the hepatic estrogen receptor via an mTOR mechanism. IGF-1 serves as an indicator of the animal’s nutritional status to the reproductive system, influencing uterine activity and the regulation of the estrous cycle [9,14]. The effectiveness of these interventions is evident, for instance, in the inoculation of taurine in mice, which stimulated estrogen synthesis in the ovaries by regulating microRNA-7a2 expression in granulosa cells [15].

Additionally, supplementing the diet of rats with methionine promoted several enhancements in reproductive activity, such as follicular growth through the regulation of genes, including growth differentiation factor 9 (GDF9) and bone morphogenetic protein 15 (BMP15). It also increased estrogen levels during the estrous cycle through mechanisms involving AA transporters, DNA methyltransferases, and cystathionine gamma-lyase as well as the number of embryos implanted in early gestation [16].

Another strategy to circumvent the ruminal manipulation mechanism is to use precursors for AA synthesis in feed, such as creatine and its precursor, guanidine acetic acid (GAA). Creatine can be synthesized endogenously or acquired through the diet and is captured via the creatine transporter (SLC6A8) [17]. Its synthesis involves two enzymes: L-arginine/glycine amidinotransferase (AGAT), which catalyzes the production of GAA, and guanidine acetate N-methyltransferase (GAMT), which catalyzes the methylation of GAA to produce creatine and S-adenosyl homocysteine [18]. Intracellular creatine is stored as phosphocreatine, and when needed for cellular processes, the cytosolic isoforms of creatine kinase (CK), specifically brain-type (CKBB) and muscle-type (CKMM), hydrolyze the bond between creatine and the phosphate group, rapidly forming ATP and creatine [19]. This reaction plays a role in the metabolism of tissues with high energy demands, such as skeletal muscle [20], and the ovaries during oocyte maturation [18,21]. Guanidine acetic acid has a well-established bibliography as a feed additive for swine and poultry, aimed at improving feed intake, growth performance, feed efficiency, and meat quality, with minimal cost and environmental impact [20]. The GAA market is currently experiencing significant expansion. In 2022, its production value was USD 37 million, and it is estimated to reach USD 53.02 million by 2031, with an annual growth rate of 4.1% [22].

In ruminants, the potential for using GAA has been explored recently, as ruminal supplementation with 0.5 g/kg or 1.0 g/kg of GAA in the diet for 100 days demonstrated positive effects on creatine degradation rate in cattle, approximately 50%, allowing half of this component to be metabolized at the intestinal level [23]. Consequently, GAA offers new perspectives on its use in ruminant feeding. In beef cattle, ref. [24] reported improved performance and diet digestibility with GAA doses of 0.6 g and 0.9 g/kg of dry matter in the diet during 90-day trials, and in lambs, metabolism and muscle activity and growth were improved [25]. However, despite these promising results in terms of production and nutrition, the extent to which GAA, as a creatine precursor, can influence reproductive function remains unclear. Recently, ref. [26], using 0.2% GAA in the diet of Brahman cows during the final 90 days of gestation, observed no effects on offspring performance, but an increase in placental vascularization was noted. The European Food Safety Authority [27], in a recent comprehensive review on the use of GAA in animal nutrition, pointed out the lack of available experimental reference data on the role of GAA in reproductive performance in ruminants. Based on the scenario described above, the main objective of this study was to fill this gap by providing information in sheep on the impact of dietary GAA supplementation on ovarian response. Our hypothesis postulates that ruminal escape of GAA is effective and, when administered in high doses for a short period, stimulates ovarian activity. Against this backdrop, this study aimed to provide an initial assessment of the effects of dietary GAA supplementation on ovarian response in sheep. Our hypothesis posits that the ruminal escape of GAA is effective and, when administered in high doses for a short period, stimulates ovarian activity.

Therefore, the objective of this investigation was to administer GAA as a dietary supplement to sheep at high doses during a hormonal protocol in preparation for mating and to evaluate its impact on the metabolic profile, follicular growth, ovarian and intraovarian blood perfusion, and the reproductive response post mating.

## 2. Materials and Methods

### 2.1. Location, Animals, Pre-Experimental Conditions, and Experimental Design

The study was conducted at the farm of the School of Veterinary Medicine at Ceará State University, Brazil, in the equatorial zone (4°2′23″ S and 38°38′14″ W). Twenty adult, multiparous Santa Inês sheep from the school’s flock were selected for the trial. During the pre-experimental period, the animals were housed in a common pen where health and reproductive controls were performed according to Fernandes et al. [28].

Twenty adult, non-lactating, non-pregnant, and between second and third parity Santa Inês sheep from the school’s flock were selected for the trial. Before the experiment, animals were submitted to a 30-day housing adaptation after receiving endo- and ectoparasite treatments and vaccinated against clostridiosis. Throughout the pre-experiment, cyclicity and ovarian function were monitored by ultrasound examinations and sexual receptivity to fertile mature ram according ref to [28]. The ewes were then grouped based on their body mass indexes (BMIs) in collective covered pens with concrete floors, and received water and mineral salt ad libitum to adapt to the experimental feeding regime. The diet consisted of a total mixed ration (TMR) composed of fresh, chopped elephant grass (*Pennisetum purpureum Schum*) and concentrate feed (50% corn, 5% soybean meal, 40% wheat bran, and 5% vitamin/mineral supplement), provided in amounts to meet the nutritional requirements of breeding adult sheep [29]. The feed was provided in two daily meals at 08:00 and 15:00, and feed intake was monitored during the experimental period. The animals were weighed, and adipose and muscle masses were measured by ultrasonography to assess subcutaneous fat thickness of the loin, depth of loin muscle, and perirenal fat according to Morales-Martinez et al. [30] and Wang et al. [31]. The sheep’s BMIs were calculated as follows: BMI = ((Body weight [kg]/Height at withers [m]/Body length [m])/10). The overall means (±standard deviation) for age, body weight, height at withers, and body length were 2.7 ± 0.9 years, 43.0 ± 5.8 kg, 66.8 ± 3.6 cm, and 65.6 ± 4.0 cm, respectively. These two anatomical areas and BMI index were used because of their close relationship with the body condition of sheep [30,32].

**Figure 1:**
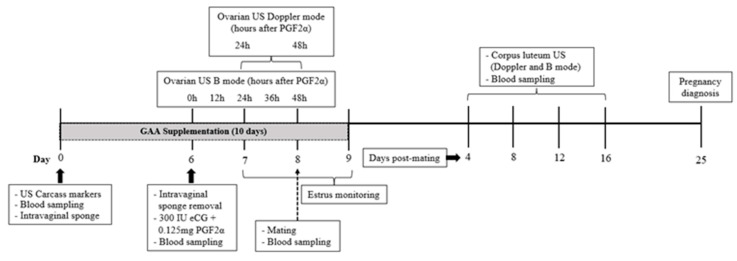
Timeline of experimental stages, including guanidine acetic acid (GAA) supply and hormonal protocol.

All ewes were prepared for mating by a hormonal treatment to synchronize estrus and follicular wave according to Casali et al. [33], using a protocol illustrated in Figure 1. Briefly, before mating, an intravaginal sponge impregnated with 60 mg of medroxyprogesterone acetate (Progespon^®^, Zoetis, São Paulo, Brazil) was inserted into the cranial portion of the vagina of each ewe. After six days, the sponge was manually removed, and 300 IU of equine chorionic gonadotropin (eCG) (No-vormon^®^, Zoetis, São Paulo, Brazil) and 0.125 mg of prostaglandin (Sincrocio^®^, Ourofino, São Paulo, Brazil) were administered intramuscularly. Twenty-four hours after prostaglandin application, estrus detection began and was conducted every 4 h for three days using a ram. Twelve hours after the onset of estrus, three matings were performed for each ewe at 12 h intervals using a Dorper ram of proven fertility.

Twenty-four hours after the prostaglandin application, estrus detection began and was conducted every 4 h for three days using a ram. Twelve hours after the onset of estrus, three matings were performed for each ewe at twelve-hour intervals, using a Dorper ram of proven fertility. Pregnancy diagnosis was performed by ultrasonography 25 days after mating.

At the onset of the hormonal protocol, the animals were divided into two nutritional treatment groups: the group fed the baseline diet (BSD; n = 10) and the GAA group (GAAD; n = 10), where animals received the baseline diet supplemented daily with 0.9 g/kg of dietary DM of guanidine acetic acid for 10 days, from the first day of the synchronization protocol until mating (Figure 1). Guanidine acetic acid in powder form (GuanAMINO^®^, Feed Grade 96.0%, Evonik Leading Beyond Chemistry, Hanau, Germany) was distributed in equal doses in each of the two daily meals.

Table 1 displays the sheep traits at the start of the experimental trial. The groups were homogeneous (*p* > 0.05) in terms of body mass index, thickness of lumbar and renal subcutaneous fat, and muscle mass as measured by loin depth.

### 2.2. Assessment of Ovarian Blood Flow and Intraovarian Blood Perfusion Area

Ovarian and intraovarian blood perfusion were measured 24 h and 48 h after prostaglandin administration (Figure 1), using an ultrasound device equipped with color Doppler functionality (model Z5 Vet; Mindray Bio-Medical Electronics Co., Shenzhen, China) and a transrectal linear transducer at a frequency of 5.0 MHz. The equipment settings of pulsed repetition frequency (PRF) at 1.0 kHz, depth at 6.5 cm, and color gain at 60% were consistently maintained during the evaluations. Ovarian blood perfusion values were obtained using the pulsed Doppler function, and following the methodology described by [34], each ovarian artery (right and left) was initially identified using the color Doppler function, which displayed the artery next to the ovarian vein as a pulsating, colored point on the ovarian pedicle. Once the artery was visualized, the pulsed Doppler function was activated with a 1.0 mm Doppler gate and a 30° insonation angle to obtain blood flow waveforms. The systolic peak (SP), diastolic peak (DP), and systole/diastole ratio (S/D) values were automatically calculated by the equipment from two spectral Doppler waveforms.

Intraovarian perfusion, reflecting the vascularization of growing follicles, was identified using ultrasound videos taken from the right and left ovaries with the color Doppler function activated. These videos were later used to calculate the color Doppler area of each ovary, following the methodology described by [35]. Using ImageJ^®^ software (Version 1.54g, National Institutes of Health, Millersville, PA, USA) calibrated for this evaluation, color Doppler images of the cross-section of each ovary were used to determine the total ovarian area (TA) and the color Doppler area (DA), which were delineated manually using the software’s freehand selections feature. From the TA and DA values, the percentage of the intraovarian Doppler area (DA/TA × 100) for each ovary on each day of evaluation was calculated.

### 2.3. Follicular Dynamics, Corpus Luteum Growth, and Blood Perfusion Area

For three days, every 12 h starting on the day of prostaglandin application (Figure 1), follicular dynamics were analyzed by ultrasonography (model Z5 Vet; Mindray Bio-Medical Electronics Co., Shenzhen, China) using a transrectal linear probe with a frequency of 5.0 MHz. Each ovary was evaluated, and videos were recorded for later measurement using ImageJ^®^ software (Version 1.54g, National Institutes of Health, Millersville, PA, USA).

Every four days, from day four to sixteen after mating (Figure 1), the diameter and Doppler area of the CL were measured to monitor the growth and vascularization of the luteal tissue [36]. Measurements were conducted using B-mode and color Doppler ultrasonography (model Z5 Vet; Mindray Bio-Medical Electronics Co., Shenzhen, China), equipped with a transrectal linear transducer with a frequency of 5.0 MHz. The settings (color gain = 60%, PRF = 1.0 kHz) were consistently maintained across all evaluations. Videos of both ovaries were recorded to identify the luteal structures. During the ultrasound evaluation, the CL was initially visualized using the B-mode function, followed by activation of the color Doppler function. Subsequently, with the previously calibrated ImageJ^®^ software (Version 1.54g, National Institutes of Health, Millersville, PA, USA), the mean diameter of the CL (average of the vertical and horizontal dimensions) was calculated. The color Doppler area was obtained by employing the software’s freehand selections function using images of the cross-section of the CL at its largest diameter. The CL and its colored areas were manually delineated to determine the total area of the CL and the luteal Doppler area, data which were used to calculate the percentage of the Doppler area.

### 2.4. Blood Sampling and Metabolite Assays

Blood samples were collected on days 0 (at prostaglandin application), at mating, and on days 4, 8, 12, and 16 post mating (Figure 1). Collections were conducted in the morning while the animals were fasting, via jugular venipuncture into 4 mL vacuum tubes containing lithium heparin anticoagulant (FIRSTLAB^®^, Disera Tıbbi Malzeme Lojistik San. Tic. A.Ş, Izmir, Turkey). The blood was then centrifuged at 3000 rpm for 10 min to separate the plasma, which was stored at −20 °C for later analyses of glucose, cholesterol, triglycerides, total proteins, creatinine, and urea using an automated biochemical analyzer (Mindray^®^ BS 120, Mindray Biomedical Electronics Co., Shenzhen, China). The analyses were conducted using commercial kits (Bioclin^®^, Quibasa, Minas Gerais, Brazil), with the sensitivities of the kits for glucose, cholesterol, triglycerides, total protein, creatinine, and urea being 1.31 mg/dL, 0.67 mg/dL, 2.58 mg/dL, 0.043 g/dL, 0.0395 mg/dL, and 1.514 mg/dL, respectively.

### 2.5. Statistical Analysis

Statistical analyses were conducted using Statistica Software version 13.4.0.14 (2018; TIBCO Software, Inc., Palo Alto, CA, USA). Data were initially verified for normality using the Shapiro–Wilk test. If this condition was not met, a log10 transformation was applied. Data were analyzed using the General Linear Model (GLM) procedures of ANOVA. The factors included in the model were diet (BSD and GAAD), the interval of the sample (time), and interactions. All pairwise comparisons were performed using the Newman–Keuls post hoc test. For pregnancy rate, twin rate, and pregnancy failure rate, the effect of the diet was analyzed using the Mann–Whitney test.

## 3. Results

### 3.1. Feeding Response and Peripheral Metabolite Levels

Table 1 depicts the response of the experimental groups to the diets, expressed as dry matter (DM) intake relative to the animals’ live weight and in kilograms per animal. Neither of these parameters showed differences between the diets (*p* > 0.05) or any interaction (*p* > 0.05) between the diets and administration time (Table 1). The total average consumption was, respectively, 1.0 kg of DM/ewe, approximately 2.3% of body weight.

The metabolic markers glucose, cholesterol, and triglycerides also showed no differences between the nutritional treatments (*p* > 0.05), nor was there any interaction (*p* > 0.05) between groups and measurement intervals (Table 1). The overall means were for glucose, cholesterol and triglycerides were, respectively, 59.3 mg/dL, 54.1 mg/dL, and 24.7 mg/dL.

**Figure 2:**
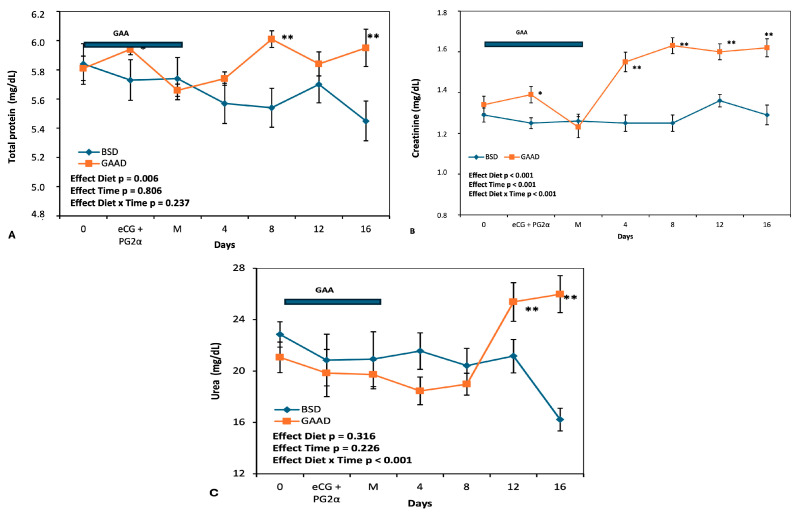
Peripheral protein (**A**), creatinine (**B**), and urea (**C**) levels measured during the experimental period in ewes fed a baseline diet (BSD) or a diet supplemented with guanidine acetic acid (GGAD). *p*-values of the ANOVA effects for diet, sampling interval (time), and interaction are given. Values are represented as means ± SEM. * *p* < 0.05 differences between diet groups. ** *p* < 0.01 differences between diet groups. Bar represents the GAA supply period.

Figure 2A–C display the values of total protein, creatinine, and plasma urea. All three markers exhibited changes during the experimental interval. Total protein was higher (*p* < 0.05) in the GAA group compared to the control at the time of PGF2α + eCG administration, six days after the start of GAA supplementation, and on days 8 and 16 post mating (*p* < 0.01) (Figure 2A). A significant interaction (*p* < 0.001) was observed between the diet factor and the measurement interval for creatinine concentrations (Figure 2B), with higher values (*p* < 0.05) in the GAA group compared to the control on the day of PGF2α + eCG administration and on days 4, 8, 12, and 16 post mating (*p* < 0.01) (Figure 2B). An interaction (*p* < 0.001) between diet and time for plasma urea was also noted (Figure 2C) due to a significant increase (*p* < 0.01) in the metabolite in animals supplemented with GAA from day 12 post mating.

### 3.2. Ovarian Artery Blood Flow and Intraovarian Blood Perfusion Area

**Figure 3:**
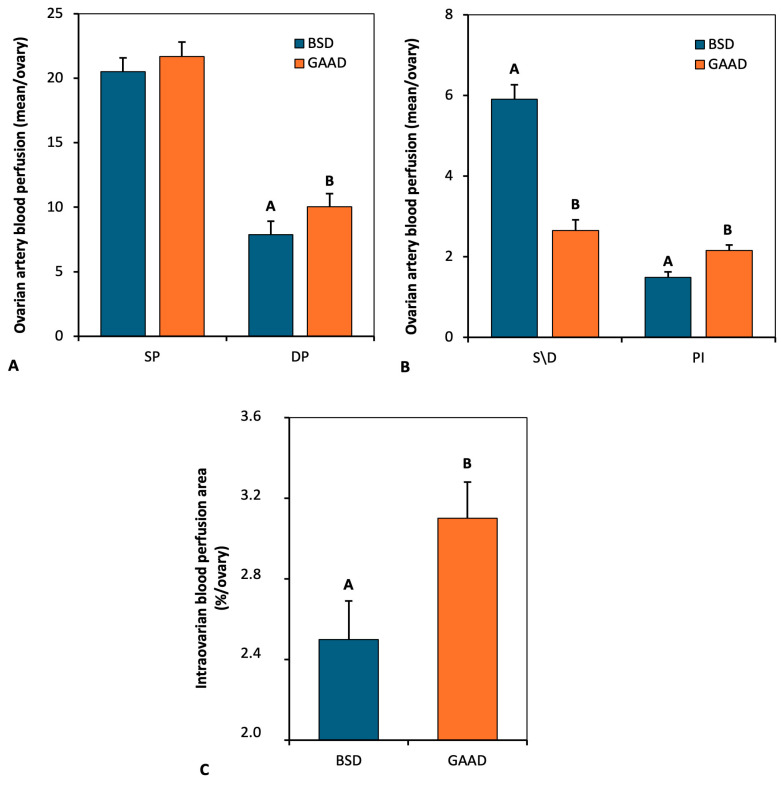
Systolic peak (SP) and diastolic peak (DP) (**A**); systolic/diastolic index (S/D) and pulsatility index (**B**) as measured in ovarian artery with doppler ultrasonography 24 h and 48 h after prostaglandin application in ewes fed a baseline diet (BSD) or a diet supplemented with guanidine acetic acid (GAAD); Intraovarian blood flow perfusion area as measured by Doppler ultrasonography 24 h and 48 h after prostaglandin application in ewes fed BSD or GAAD (**C**). Values are represented as means ± SEM. A and B: *p* < 0.05 differences between diet groups.

Figure 3A,B document the Doppler parameters of blood flow in the ovarian artery 24 h and 48 h after PGF2α + eCG administration. There was an increase (*p* < 0.05) of 27% in the diastolic peak (Figure 3A) and 45% in pulsatility (Figure 3B) in the GAA diet, while the systolic peak did not differ (*p* > 0.05) between the groups (Figure 3A). The ratio between the systolic and diastolic peaks (S/D index; Figure 3B) was lower (*p* < 0.05) in the GAA group compared to the control. Figure 3C depicts the area of intraovarian blood perfusion measured by Doppler 24 h and 48 h after PGF2α + eCG administration. Animals supplemented with GAA showed a 24% (*p* < 0.05) increase in blood perfusion area.

### 3.3. Follicle Turnover

**Figure 4:**
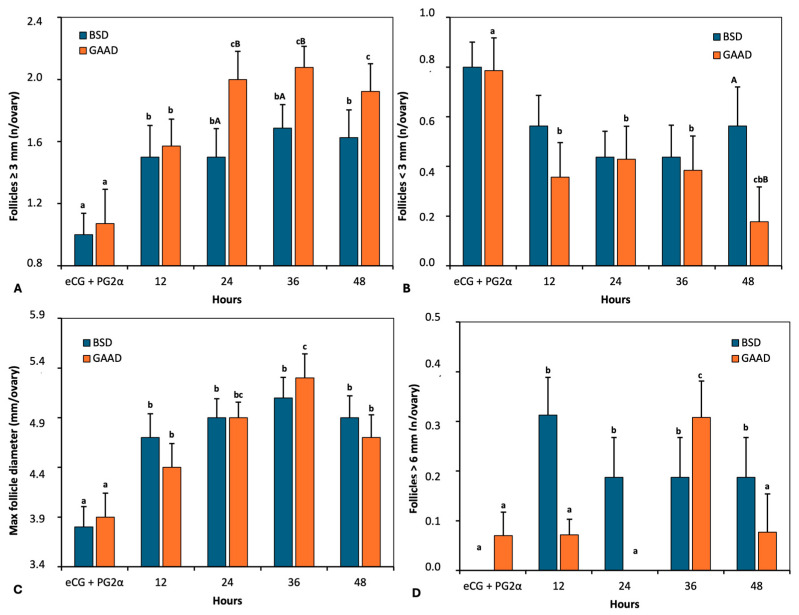
Number of follicles ≥3 mm (**A**) and <3 mm (**B**), maximum follicular diameter (**C**), and number of follicles >6 mm (**D**) as measured by ultrasonography over 48 h from prostaglandin application in ewes fed a baseline diet (BSD) or a diet supplemented with guanidine acetic acid (GAAD). Values are represented as means ± SEM. a, b, and c: *p* < 0.05 differences between time intervals. A, B *p* < 0.01 differences between diet groups for each interval.

Both groups recorded a significant increase in the number of follicles with a diameter ≥3 mm up to 24 h after PGF2α + eCG administration (Figure 4A). The maximum value of this follicular class was reached 36 h after PGF2α + eCG. During these intervals (24 h and 36 h), the GAA diet recorded a higher number (*p* < 0.05) of these follicles than the control (Figure 4A). Throughout the measurement interval, a significant reduction (*p* < 0.05) was also observed in follicles with a diameter <3 mm in the GAA group (Figure 4B). In the control group, on the contrary, no differences (*p* > 0.05) were observed between the measurement intervals. After 48 h of PGF2α + eCG, the two diets differed significantly (*p* < 0.05; Figure 4B), with a 67% reduction in the GAA group compared to the control (0.87 follicles/ovary vs. 0.28 follicles/ovary; *p* < 0.05).

Regarding the maximum follicular diameter, there was a significant increase (*p* < 0.05) up to 36 h after PGF2α + eCG administration in the GAA diet (Figure 4C), while for the BSD, no differences (*p* > 0.05) were observed between intervals after 12 h of PGF2α + eCG. Figure 4D illustrates the number of follicles with a diameter > 6 mm. In the GAA diet, there was an increase (*p* < 0.05) in this follicular class 36 h after PGF2α + eCG, while for the BSD group, the peak of these follicles was recorded 12 h after PGF2α + eCG.

### 3.4. Reproductive Outcome

Table 2 presents the results of the estrus induction treatment and the reproductive response after mating. All animals exhibited estrus. The onset and duration of estrus showed no differences (*p* > 0.05) between the diets. The overall means were, respectively, 32.5 h and 42.5 h.

After mating, no differences (*p* > 0.05) were observed between the groups in relation to the number of CL, the CL area, and its blood perfusion area (Table 2). Despite this, in both diets, the CL area and its blood perfusion showed a significant increase depending on the measurement interval (effect time *p* < 0.001; Table 2). These parameters reached their peak values at 12 days post mating. Nutritional treatments also did not show any differences (*p* > 0.05) in relation to pregnancy and twin births rates, prolificacy, and early pregnancy failure rate (Table 2).

## 4. Discussion

The findings of this study support the initial hypothesis that supplementation with GAA stimulates ovarian function in sheep. The results demonstrate how the application of GAA at selected dosages and timing was targeted to optimize follicular growth and ovarian vascularization but did not enhance the animals’ ovulatory response or subsequent pregnancy rates, which remained similar to those of the control group.

The substantial increase in plasma proteins and urea, along with the rise in creatinine levels observed in this study, confirmed that the dosage of GAA used was effective in promoting greater protein availability in the form of creatine in the animals.

As we know, creatine can be synthesized endogenously or acquired through the diet. In endogenous synthesis, the enzyme L-arginine/glycine amidinotransferase (AGAT) catalyzes the formation of GAA and ornithine from arginine and glycine. The enzyme guanidinoacetate N-methyltransferase (GAMT) then methylates GAA, producing creatine and S-adenosyl homocysteine. In humans, the kidneys are the primary site of GAA formation, with GAA being released and methylated predominantly by GAMT in the liver, pancreas, and, to a lesser extent, in the kidneys to produce creatine [37]. From the diet, plasma creatine is absorbed by intestinal and extraintestinal cells via the creatine transport protein (SLC6A8) [38], whose activity depends on several factors, such as substrate concentration and Na^+^ transmembrane gradients [39]. At the mitochondrial level, in a reaction catalyzed by ubiquitous mitochondrial creatine kinase (uMt-CK) or creatine kinases (CK) associated with glycolytic enzymes, both creatine molecules synthesized by the body and absorbed from the diet are phosphorylated to form adenosine diphosphate (ADP) and phosphocreatine [40]. In ruminants, approximately 50% of dietary GAA undergoes ruminal degradation and is utilized by microorganisms [23]. In the small intestine, dietary creatine is absorbed through the apical membrane of enterocytes and enters the portal circulation, where it is freely transported in the blood and rapidly absorbed by various tissue cells [37].

The GAA dosage used (0.9 g/kg DM), as reported [24], is among the most effective in terms of digestibility and performance for Angus bulls. It increased plasma creatine levels, promoted greater DM intake, and enhanced average daily gain in addition to improving the digestibility of DM, organic matter, NDF, and ADF. It also elevated the concentration of volatile fatty acids in the rumen, leading to the conclusion that adding GAA to the diet for 90 days improves not only growth performance but also nutrient digestion. The substantial increases in plasma protein and urea, along with the rise in creatinine observed in the current study, confirmed that the GAA dosage was effective in boosting protein availability in the form of creatine in the animals. Increased dietary creatine intake enhances its plasma and tissue concentrations, including in skeletal muscle, brain, and heart [32,41].

Creatine is converted to creatinine through the spontaneous loss of a water molecule [42]. However, according to [17], less than 2% of the creatine available in the body is irreversibly converted to creatinine, indicating that the majority of creatine used for ATP production is recycled and restored in the CK circuit as phosphocreatine [37]. Therefore, the increase in plasma creatinine indicates that the GAA diet enhanced the production and availability of creatine to meet bioenergetic demands. Additionally, it was described that increased creatine availability spares arginine, glycine, and methionine for use in other vital metabolic pathways, such as protein, nitric oxide, and glutathione synthesis [42], which may explain the increase in plasma protein levels in the GAA-supplemented group.

After the administration of eCG, animals supplemented with GAA exhibited a pronounced depletion of small follicles (<3 mm), coupled with a simultaneous increase in the number of growing follicles (>3 mm). These events are anticipated as part of the follicular wave growth induced by gonadotropin administration. Blood flow is the main vehicle for delivering nutrients and functional signals to the ovary, and the processes of follicular activation, growth, and maturation depend on extensive intraovarian microvascularization to supply the follicles with nutrients, oxygen, and gonadotropic and steroid hormones [43].

Following the application of eCG, both local blood flow and plasma concentrations of LH and estradiol rise simultaneously [44], enhancing the production of steroids and vasoactive substances to support follicle growth. To increase blood flow, estradiol activates endothelial nitric oxide synthase (eNOS) through mechanisms involving the membrane-associated estrogen receptor (ER), heat-shock protein 90 (HSP90), and AMP-activated protein kinase (AMPK) [45,46], which promotes dilation of the vascular endothelium [44,47]. Nitric oxide, a vasodilator and angiogenic factor formed from L-arginine, plays a key role in regulating blood flow [37,48]. Thus, the enhanced ovarian microvascularization at this stage ensures the delivery of gonadotropins necessary for follicular growth. The results indicated that in the GAA group, follicular growth was not only more efficient but was also accompanied by improved vascularization of the ovarian artery and the intraovarian microcapillary system. As previously noted, creatine supplementation can increase plasma concentrations of other AAs such as arginine [26], which are utilized in additional metabolic pathways, including the phosphorylation of endothelial NO proteins [42].

In the ovaries, the origin of creatine remains uncertain, although high expression of the creatine transporter gene (SLC6A8) has been observed in rat ovaries [49], suggesting that plasma creatine is transported into ovarian cells. Additionally, evidence indicates that the creatine metabolic pathway may also be present in the ovarian stroma, as gene expression of the GAMT and AGAT enzymes has been detected in these cells in female ovaries [50]. The gonadotropic effects of creatine are still not well understood. However, creatine has been identified in human follicular fluid (FF) [51], and studies in mice have shown an increase in creatine concentrations in FF around ovulation [50]. Follicular fluid is derived from blood plasma through intraovarian vasculature and from secretions synthesized in the follicle wall and granulosa cells [52]. The expression of CK pathway genes has been found to be elevated in cumulus cells [53]. Ref. [54] proposed that the rise in creatine levels in FF is due to an increased capacity for creatine synthesis within the follicular microenvironment itself, as granulosa cells, which provide nutritional support for oocyte maturation, showed expression of the AGAT and GAMT genes, especially with eCG stimulation close to ovulation. In cattle, the presence of GAA and an increase in creatine concentrations were observed in the maturation medium of cumulus–oocyte complexes (COCs) [55].

It is known that communication within COCs is bidirectional, as the structural organization of the cells allows for the transport of molecules in both directions, and signals originating from the oocyte can interact with receptors on cumulus cells, modulating their function [56]. In bovine oocytes, gene and protein expression of ubiquitous uMt-CK and CKBB activity increased, with higher gene expression of CK in immature oocytes [21], ensuring the storage of phosphocreatine to help maintain intracellular ATP levels. Depending on cellular need and the available levels of intracellular ADP/ATP, creatine phosphorylation is reversed according to the proportion of CK isoforms in the cytosol, which hydrolyze the bond between creatine and the stored phosphate group, regenerating ATP [32]. Among the CK isoforms, the most common is the brain type (CKBB), but the muscle type (CKMM) has also been identified in the mouse oocyte [54]. Therefore, when CK isoforms and components of creatine metabolism are present in the cell, this circuit produces ATP rapidly, meeting the high bioenergetic demands of the tissue [40], as is the case during ovulation. In mice, gene expression of CKMM and CKBB activity has been shown to increase during oocyte maturation and the early embryonic stages [49,54].

One of the main findings of this study was the observable effect of creatine on the ovaries in sheep after a brief period of GAA supplementation in the diet. As previously described, the dosage selected for this study was based on the best results available from trials on ruminants that measured productive and nutritional parameters. A primary challenge was thus to determine whether this model could be effective in short-term reproductive protocols. The existing literature on GAA use in ruminants and other species predominantly focuses on long-term trials (over 10 weeks) and primarily examines productive response parameters [27,57]. Recently, ref. [26] tested a 0.2% GAA supplementation in cattle over a 12-week trial during gestation, finding no differences between groups in serum and uric creatine concentrations or offspring performance. However, they observed increases in serum NO, placental vascularization, and plasma concentrations of arginine, ornithine, citrulline, and tyrosine as well as AGAT activity in the liver.

The authors have speculated that the decrease in DM intake might be linked to the animals’ mechanisms for regulating their energy intake, as GAA also increases the energy concentration of the TMR. Another recent study by [58], testing various concentrations (0.2%, 0.4%, 0.6%, and 0.8%) of GAA in the diet of fistulated female cattle over 48 h of in vitro fermentation, demonstrated a significant increase in microbial protein and propionate in the rumen. This study also noted a reduction in isovalerate and an increase in the bacterial communities *Bacteroidota*, *Prevotella*, and *Prevotellaceae_UCG-001* with 0.8% GAA. Additionally, metabolic pathways related to bile secretion, digestion, and protein absorption were enhanced, leading to the conclusion that GAA improves ruminal fermentation parameters and increases the relative abundance of bacteria associated with the degradation of non-fibrous substances, thus providing more energy to the animal.

Our results showed that GAA exerted its effects on the follicular environment within a short timeframe, in this instance, after only 10 days of supplementation. Nonetheless, the chosen protocol did not effectively alter the ovulatory rate or the quality of the corpus luteum. These outcomes were partially anticipated, as it is well recognized that follicular growth, oocyte competence, and embryonic development are processes that have distinct nutritional requirements.

Amino acid metabolism varies with embryonic stage; for example, glutamine, which is prevalent in proliferating somatic cells, is abundant in two- and four-cell bovine embryos before decreasing inversely with glucose uptake and then increasing again during blastocyst expansion due to heightened protein synthesis [59]. Additionally, selenocysteine, a component of glutathione peroxidase, is found in oocytes and early embryos, and although it enhances blastocyst rates, its specific role in mammalian oocytes or embryos has yet to be fully understood [59]. Maternal nutrition is known to influence the physiological mechanisms required for the acquisition of oocyte competence and embryonic development. The oocyte requires substantial energy during its development in preparation for the bioenergetic demands of maturation and fertilization. Therefore, the periconceptional period is critical, as nutrition can induce various effects, primarily through epigenetic modifications and mitochondrial deficiencies [60]. During oocyte maturation, components such as RNA and proteins accumulate to support the future development of the embryo until the activation of the embryonic genome. Consequently, during these processes, various molecules, including amino acids, pyruvate, fatty acids, cytokines, and growth factors, are modulated by nutritional intake [61].

Oocytes metabolize glucose via glycolysis, pentose phosphate pathways, and the tricarboxylic acid cycle, contributing to ATP production and NADPH supply [62]. However, amino acids can also be metabolized by the tricarboxylic acid cycle to produce ATP. These nutrients are utilized by the cumulus–oocyte complex and enhance developmental competence for the blastocyst stage [63]. Therefore, limitations in amino acids and proteins can impair the energy metabolism of reproductive cells.

According to [61], protein restriction can affect the ultrastructure of oocyte mitochondria. A low-protein diet influences the expression of genes that mediate mitochondrial fusion, which can lead to defective mitochondrial biogenesis and dysregulation of apoptosis in oocytes [61]. Additionally, the metabolism of protein components varies with the oocyte stage, as immature bovine oocytes exhibit higher expression of creatine kinase compared to mature oocytes. This suggests that creatine kinase activity is integral to maintaining intracellular ATP levels, ensuring successful maturation [18].

Moreover, nutrient utilization changes after fertilization and the onset of embryonic development. In embryos, ATP production occurs to provide energy for cell growth and development, with glucose as the primary energy source, although other nutrients such as amino acids are also important [62]. Regarding carnitine metabolism, it occurs differently in oocytes and embryos. Ref. [64] noted that cumulus cells do not express transcripts for enzymes involved in carnitine synthesis, indicating that oocytes rely on carnitine uptake from follicular fluid. In embryonic metabolism, however, transcripts of carnitine palmitoyltransferase 1B were temporarily detected at the zygote stage in mice and reappeared at the morula and blastocyst stages [65]. Another observation from the same study shows that carnitine palmitoyltransferase 2 transcripts decreased post fertilization to undetectable levels but were present again during the morula stage, concurrent with increased oxygen uptake and fatty acid oxidation [65].

## 5. Conclusions

The inclusion of guanidine acetic acid in the diet of ewes at a dose of 0.9 g/kg of dry matter for 10 days before mating significantly altered the ovarian blood supply and improved follicular growth, but it did not affect the pregnancy rate or prolificacy. Therefore, we intend to conduct further prospective studies on the effects of guanidine acetic acid in sheep, exploring new doses and application times to establish effective supplementation protocols that support its adoption within the proper period for the breeding of this species.

## Figures and Tables

**Table 1:** Body mass index, fat loin subcutaneous thickness, kidney fat thickness, loin depth, feed intake, and peripheral metabolite levels in ewes fed with baseline diet (BSD) or supply with guanidinoacetic acid (GAAD).

Parameter	Diet			*p*-Value		
	BSD	GAAD	SEM	Diet	Time	D × T
Body and carcass marker *						
BMI	10.5	9.1	0.486	0.142	-	-
SLFT, mm	3.2	2.9	0.106	0.310	-	-
KFT, mm	1.3	1.4	0.025	0.130	-	-
LD, mm	23.5	22.9	0.593	0.619	-	-
Feed intake						
DMI, kg/ewe	1.0	1.0	0.012	0.567	0.800	0.965
DMI, % BW	2.4	2.3	0.028	0.569	0.782	0.984
Peripheral metabolic marker						
Glucose, mg/dL	60.0	58.4	0.554	0.146	0.224	0.562
Cholesterol, mg/dL	55.2	53.1	0.909	0.321	0.904	0.312
Triglycerides, mg/dL	25.5	24.0	0.823	0.132	0.128	0.336

* Performed at the beginning of the experiment; BW, body weight; BMI, body mass index, SLFT, subcutaneous loin fat thickness; KFT, kidney fat thickness; LD, loin depth; DMI, dry matter intake. Time: ANOVA effect for the assessment interval adopted.

**Table 2:** Estrus, corpus luteum characteristics, and pregnancy outcome in ewes fed a baseline diet (BSD) or a diet supplemented with guanidine acetic acid (GAAD).

Parameter	Diet			*p*-Value		
	BSD	GAAD	SEM	Diet	Time	D × T
N of ewes in estrus, % (n/n)	100.0 (10/10)	100.0 (10/10)	-		-	
Estrus onset *, h	33.6	31.4	2.465	0.646	-	-
Estrus length, h	46.4	38.5	2.893	0.180	-	-
CL, n/goat	1.4	1.4	0.163	0.876	-	-
CL area, mm^2^	8.1	7.3	0.339	0.079	<0.001	0.546
CL doppler area, mm^2^/ovary	1.4	1.2	0.076	0.095	<0.001	0.905
Pregnancy rate, % (n/n)	80.0 (8/10)	70.0 (7/10)	-	0.879	-	-
Twinning rate, % (n/n)	25.0 (2/8)	28.5 (2/7)	-	0.784	-	-
Litter size, n (n/n)	1.3	1.3	0.118	0.886	-	-
Pregnancy failure **, % (n/n)	20.0 (2/10)	30.0 (3/10)	-	0.823	-	-

* Interval between progesterone sponge removal and estrus onset; ** gestation failures occurred from mating to pregnancy diagnosis. CL, corpus luteum; Time: ANOVA effect for the assessment interval adopted.

## Data Availability

The data produced during the current study are available from the corresponding author on reasonable request.
